# DNA methylation in esophageal cancer: technological advances and early detection clinical applications

**DOI:** 10.3389/fonc.2025.1543190

**Published:** 2025-07-11

**Authors:** Jiyu Tang, Xiaoming Shi, Chao Song, Wenjing Zhang, Yan Yan, Linchao Dai, Di Wu, Jie Qiu, Jiahuan Liu, Tao Wang, Zuhong Lu

**Affiliations:** ^1^ State Key Laboratory of Digital Medical Engineering, Southeast University, Nanjing, China; ^2^ Jiangsu Institute for Sport and Health (JISH), Nanjing, China; ^3^ Nanjing Drum Tower Hospital Clinical College of Nanjing Medical University, Nanjing, China; ^4^ Department of Thoracic Surgery, Affiliated Drum Tower Hospital, Nanjing University Medical College, Nanjing, Jiangsu, China; ^5^ School of Biological Science & Medical Engineering, Southeast University, Nanjing, China

**Keywords:** esophageal cancer, early detection, DNA methylation biomarkers, technology of methylation, clinical application

## Abstract

Esophageal cancer (EC) is a malignant tumor with high mortality rates, where early screening and diagnosis are critical for improving patient outcomes. DNA methylation, a key epigenetic modification, has emerged as a significant biomarker for early detection of EC. The advancement in DNA methylation sequencing technologies, including first-generation and next-generation sequencing (NGS), has revolutionized the way we identify and analyze these biomarkers. First-generation sequencing, has been instrumental in identifying specific methylation sites. However, its limited throughput renders it impractical for large-scale screening of multiple samples. In contrast, NGS offers high-throughput capabilities, allowing for the simultaneous analysis of thousands of DNA fragments. NGS significantly enhances the efficiency and accuracy of DNA methylation profiling, permitting genome-wide identification of multiple methylation markers. This approach offers a promising avenue for the enhanced early detection of EC by providing a comprehensive view of the methylation landscape. The integration of NGS into clinical practice is capable of transforming EC screening by offering heightened sensitive and specific approach to identifying patients at risk. As our comprehension of the role of DNA methylation in cancer progression deepens, the development of targeted therapies based on methylation profiles may also become a reality. In conclusion, the evolution of DNA methylation sequencing technologies has unlocked new avenues for the early EC detection. While first-generation sequencing has laid the groundwork for characterizing specific methylation events, NGS has expanded the scope of screening, offering a more robust and scalable solution for identifying early-stage EC.

## 1 Introduction

Esophageal cancer (EC) ranks among the most extremely aggressive cancers worldwide, representing the seventh highest incidence and sixth highest mortality rate of all cancers (1). In 2020, 324,000 people were newly diagnosed EC and 301,000 people died of EC in China where this malignancy occurs frequently (2, 3). EC can be divided into two major histological subtypes: esophageal squamous cell carcinoma (ESCC) and esophageal adenocarcinoma (EAC), of which ESCC accounts for around 90% of all the patients (4). ESCC is predominantly linked to risk factors such as smoking, alcohol consumption, and human papillomavirus (HPV) infection. It is more prevalent in regions with high incidence rates, such as parts of China, Iran, and South Africa (1). In contrast, EAC is closely associated with gastroesophageal reflux disease (GERD) and Barrett’s esophagus (BE), a condition where the normal squamous cells of the esophagus are replaced by columnar cells. The incidence of EAC has been rising in Western countries, paralleling the increasing prevalence of obesity and GERD (5). Molecularly, ESCC and EAC exhibit distinct genetic and epigenetic alterations. ESCC often presents with mutations in *TP53*, *CDKN2A* and *PIK3CA*, while EAC is characterized by mutations in *TP53*, *SMAD4* and HER2 (6). DNA methylation patterns also differ between the two subtypes, with ESCC showing widespread hypomethylation and focal hypermethylation of tumor suppressor genes, whereas EAC exhibits more complex methylation changes, including hypermethylation of genes involved in cell cycle regulation and apoptosis (7). These differences highlight the importance of subtype-specific biomarkers for early detection and targeted therapies.

To date, EC pathogenesis remains incompletely elucidated. Approximately80% EC patients present with locally advanced or metastatic disease at diagnosis due to the absence of specific early symptoms, resulting in poor prognosis with a 5-year survival rate below 5%. In stark contrast, the 5-year survival rate of patients at early stage can reach to 95% after curable treatments (8, 9). As a consequence, early detection and intervention of EC is the most pivotal strategy to improve the prognosis of EC patients. Although upper gastrointestinal endoscopy remains the gold standard for the early EC screening in high-risk individuals, its invasiveness, inconvenience and time-consuming procedures limit the application in mass screening (10, 11). Therefore, the development of readily accessible and noninvasive approach for the early detection of EC is more effective and suitable to relieve the global EC burden.

Liquid biopsy, defined as a non-invasive diagnostic method using body fluid samples such as blood, has become the most attractive technology in the field of cancer detection and precise management. Liquid biopsy has many advantages over traditional tissue biopsy. Liquid biopsy is non-invasive, easily reproducible and provide convenient insights into tumor burden and the response to therapy. In addition, liquid biopsy enables a molecular snapshot of the primary tumor, thus minimizing bias in biopsy results due to sampling bias and intra-tumor heterogeneity (12, 13). Analytes of liquid biopsy comprise assorted cancer biomarkers, including circulating tumor cells (CTCs), DNA methylation, exosomes, microRNA (miRNA), proteins and metabolites (14, 15). Among these blood-borne analytes, DNA methylation offers several superiorities compare to CTCs and exosomes. DNA methylation is relatively easy to extract from blood with high efficiency and purity and short half-life for real-time monitoring of tumor burden, which reflects the extent of cancer. Although DNA methylation biomarkers including somatic mutations and somatic copy number variants have shown great potential in early cancer detection and diagnosis, it still faces some pivotal challenges in clinical application. The consistency of DNA methylation detection is one of the top considerations in restricting its development (16). Cancers have great spatial and temporal genetic heterogeneity and the content of DNA methylation in diverse patients at different time points varies enormously, making it difficult to formulate a unified detection standard (17, 18).

DNA methylation is one of the earliest discovered and the most extensively studied epigenetic modifications, which occurs through the addition of cytosine methyl group at the carbon-5 position by DNA methyltransferases, which plays a crucial role in a number of biological processes, such as the regulation of gene expression, the maintenance of chromatin structure, gene imprinting and the assessment of aging phenotypes (19–21). In the 1990s, Bisulfite Sequencing (BS-Seq) was established as a classical method for detecting DNA methylation, which uses sulfite treatment to convert unmethylated cytosine to uracil and then sequencing to distinguish methylated from unmethylated cytosine (22, 23). With the advent of Next Generation Sequencing (NGS), techniques for Methylation Dependent ImmunoPrecipitation followed by sequencing (MeDIP-seq) based on antibody enrichment methods and Reduced Representation Bisulfite Sequencing (RRBS) for the genome were developed, which allow analysis of methylation at the genome-wide level (24). With the development of sequencing technology, a variety of methylation sequencing technologies have emerged, such as oxidative bisulfite sequencing(oxBS-Seq) and targeted enrichment methylation site sequencing, etc. These technologies have shown their advantages in different application scenarios (25–27). Third-generation sequencing techniques, such as nanopore sequencing, offer new methods for long-read, long-length DNA methylation analysis that help directly detect methylation at the single-molecule level while preserving the long pattern of methylation information, but this technique is currently not mature (28).

A large number of studies have indicated that the occurrence and development of cancer is accompanied by the changes of DNA methylation pattern. At the initial and progressive stage of tumorigenesis, DNA methylation level has been abnormal and cancer cells are mainly characterized by genome-wide hypomethylation and abnormally hypermethylation at specific CpG Islands (7, 29). Unlike the highly individualized and heterogeneity of gene mutations, the methylation patterns of the same cell type are extremely similar in different individuals (30). Moreover, DNA methylation detection has an excellent tissue traceability, and based on the heterogeneous methylation between different tissues, DNA methylation can accurately distinguish between primary and metastatic lesions (31, 32). GRAIL has reported the evaluations and performance comparisons of several DNA-based multi-cancer early detection approaches. At a specificity of 98%, DNA methylation has the highest sensitivity for cancer signal detection (33). Another study demonstrated that targeted DNA methylation analysis could distinguish more than 50 types of cancer, including high mortality cancer and early-stage cancers for which screening guidelines lacked, with a specificity over 99% and a single false-positive rate less than 1%. The sensitivity was 43.9% for stage I-III cancers and 54.9% for stage I-IV cancers. When cancer signals were detected, the analysis which locate the tissue origin of cancer achieved 93% accuracy (34). Owing to the above points, DNA methylation has been regarded as the most promising marker for early cancer detection. Recently, a comprehensive review on DNA methylation in both benign and malignant gastrointestinal cancers highlighted the significance of DNA methylation as a biomarker in gastrointestinal cancers (35).

**Figure 1:**
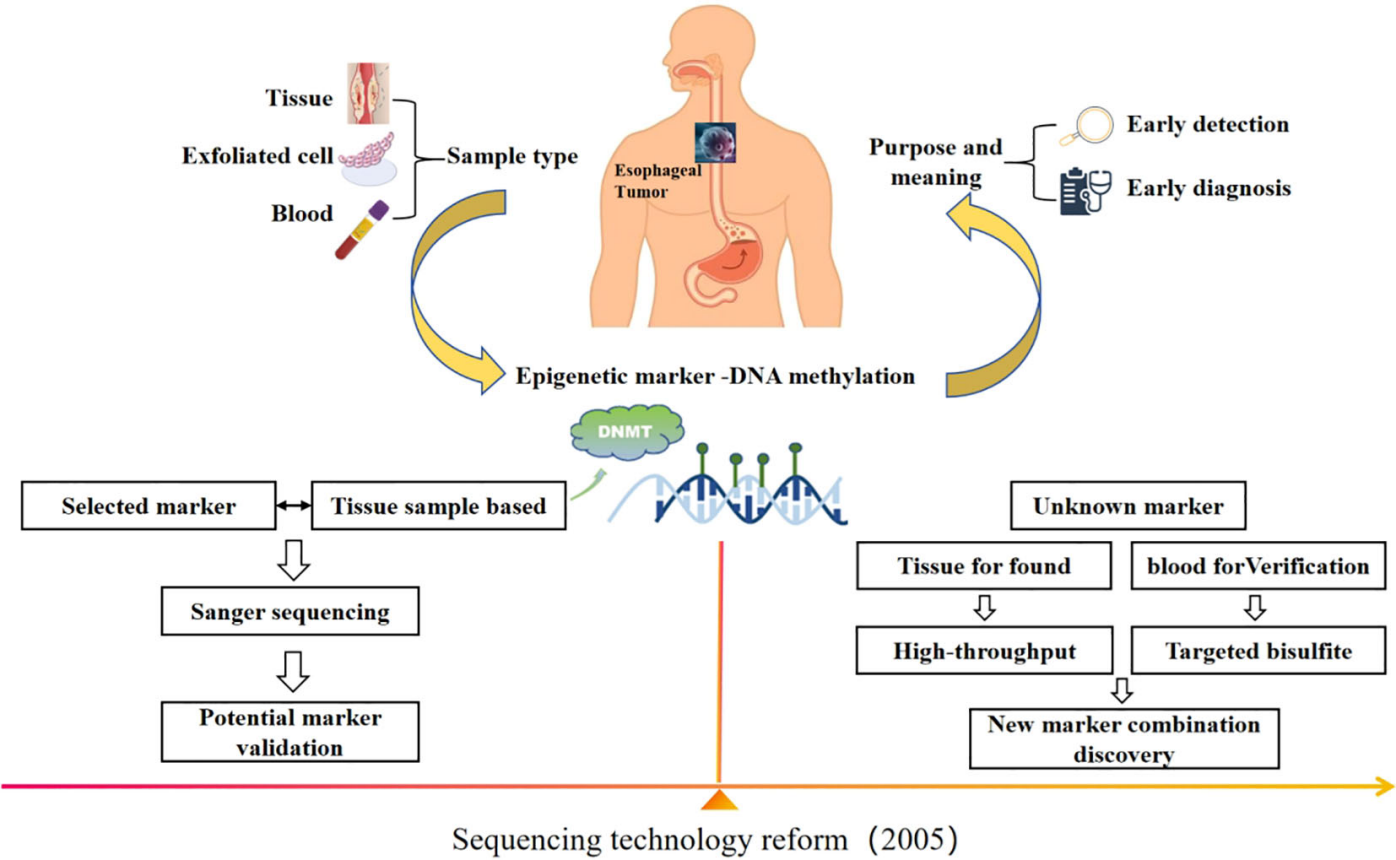
With the change of sequencing technology, DNA methylation has become a potential marker for early screening and diagnosis of EC, and the idea of site screening is different.

In this review, we first summarize the applications of DNA methylation under different sequencing techniques in early screening and diagnosis of EC, as shown in **Figure 1**. We then describe the current technologies for DNA methylation detection and analysis. Finally, we reflect on the challenges and the future directions in this field.

## 2 DNA methylation in EC

Numerous studies have demonstrated that DNA methylation is a crucial factor in the development of EC, mainly manifested in global hypomethylation and localized hypermethylation of the promoter CpG island within tumor suppressor genes (36, 37). To date, it has been identified that nearly a hundred tumor suppressor genes can be silenced due to hypermethylation in their promoter regions, which are involved in several critical pathways, including cell cycle regulation, apoptosis regulation, cell adhesion and metastasis regulation, DNA repair, WNT/β-catenin and transforming growth factor-β (TGF-β) (38, 39). In the cell cycle regulation pathway, the gene cyclin dependent kinase inhibitor 2A (*CDKN2A*) and Ras association domain family member 1A (*RASSF1A*) are frequently hypermethylated, leading to loss of function and uncontrolled cell proliferation (40, 41). In apoptosis regulation, hypermethylation of genes such as death associated protein kinase (*DAPK*) inhibits the apoptotic process and contributes to tumor progression (40). For cell adhesion and metastasis, hypermethylation and loss of expression of cadherin 1 (*CDH1*) are closely associated with tumor invasion, metastasis, and poor prognosis (42, 43). Hypermethylation of genes such as O6-methylguanine-DNA methyltransferase (*MGMT*) and mutL homolog 1 (*MLH1*) impair DNA repair mechanisms, resulting in the accumulation of genetic mutations and cancer progression (44–46). Additionally, the methylation level of anaphase promoting complex (*APC*) and zinc finger protein 382 (*ZNF382*) are associated with the differentiation level of ESCC and exert a tumor-suppressive effect by inhibiting the WNT/β-catenin pathway (47, 48). Collectively, these methylation alterations drive the development and progression of EC by disrupting key biological processes.

## 3 The development of DNA methylation detection technologies

**Figure 2:**
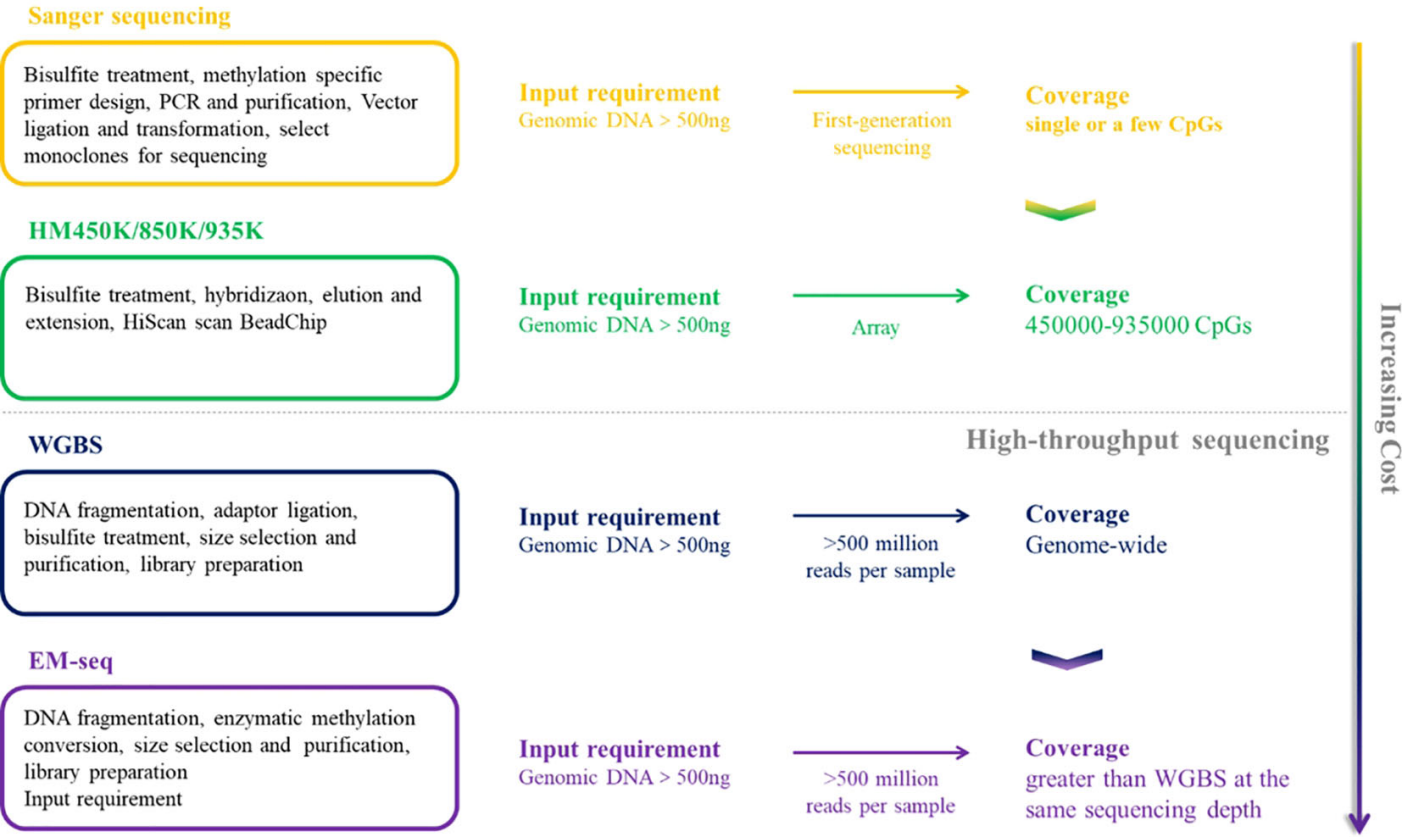
A comparative summary of the major DNA methylation detection techniques, including sanger sequencing, HM450K/850K/935K, WGBS and EM-seq.

**Figure 3:**
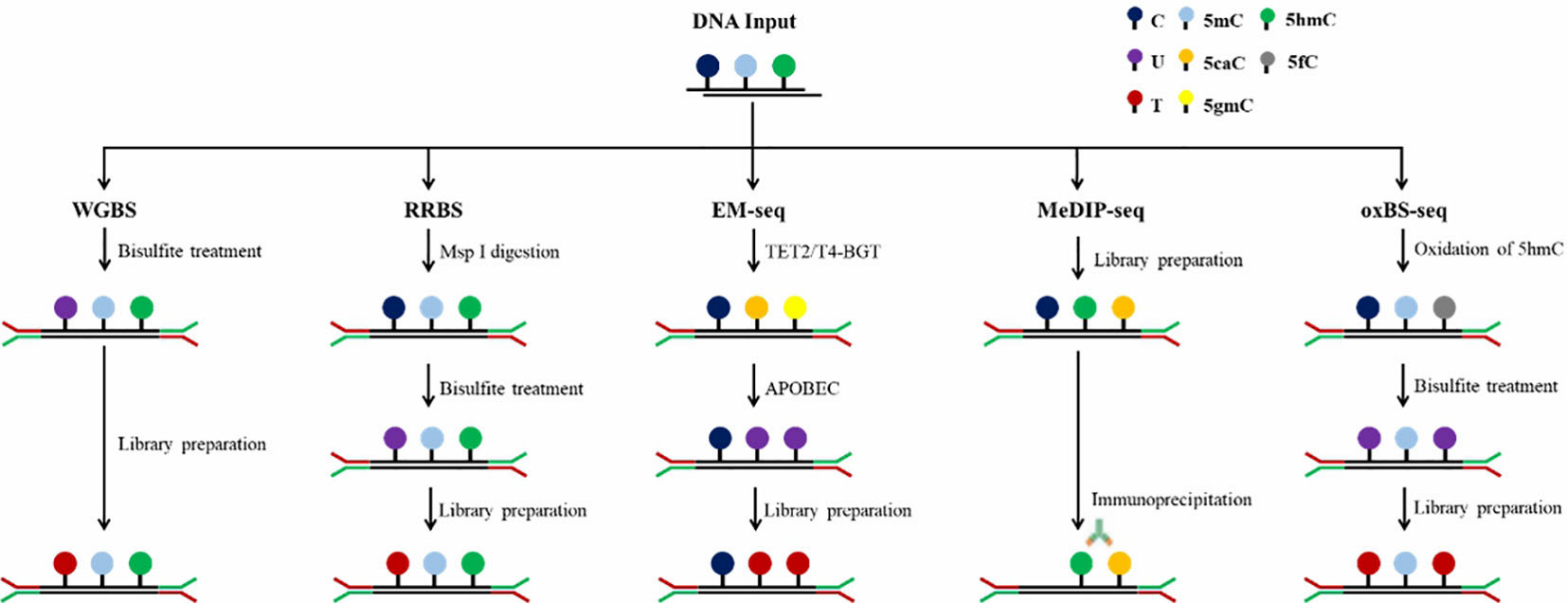
Schematic diagram of procedures of different DNA methylation technologies.

In the past few decades, the research on DNA methylation has made rapid progress, and the study of specific gene DNA methylation as a biomarker of cancer diagnosis has attracted more and more attention. A plethora of technologies has emerged to detect DNA methylation patterns, aiming to integrate this analysis into clinical practice more effectively, **Table 1** provides a comparative analysis of the characteristics, advantages and disadvantages of the current major technologies. which can be broadly categorized into two types: first-generation low-throughput detection technologies based on PCR and Sanger sequencing and high-throughput detection techniques such as methylated microarrays with pre-designed probes and genome-wide DNA methylation sequencing (49). **Figures 2** and **3** provide comparative analysis of different DNA methylation detection technologies.

**Table 1:** The comparison of major DNA methylation detection technology.

Class	Technology	Genome coverage	Resolution	Time	Advantages	Disadvantages	Cost	Applications and limitations in EC
Bisulfite-based	MSP	single or a few CpGs	Single-base	1996	ultra-low DNA input	few loci for one time and complicated primer design	Low	Capable of detecting the methylation status of specific gene in EC, but not suitable for comprehensive methylome-wide analysis
	WGBS	genome-wide	Single-base	2006	the most extensive and comprehensive genome-wide methylation profiling	relatively low sequencing depth and expensive	High	Comprehensively analyzes the whole genome DNA methylation profile of EC to reveal complex methylation patterns, but the high sequencing cost limits its application in large-scale sample analysis
	RRBS	3% of the whole genome	Single-base	2005	high CGIs coverage	restricted to regions in proximity to restrictionenzyme sites	Moderate	Lower cost than WGBS, effective for screening methylation markers in CpG islands of EC, but may overlook other significant methylation sites
	oxBS-seq	genome-wide	Single-base	2013	accurate distinction between 5hmC and 5mC	adding chemical oxidation steps make the experimental process more complicated	High	Specifically, and accurately detects the 5mC methylation status in EC, but the complex experimental and data analysis process limit its application of large clinical samples
Probe-based	450K	450000 CpGs	Single-base	2011	pre-designed probes covering hotspot methylation CpGs	low genome-wide coverage	Low	Ideal for high-precision methylation marker screening in EC, cost-effective and scalable for large clinical cohorts, but restricted to predefined sites, unable to detect new methylation sites
	850K	850000 CpGs	Single-base	2016				
	935K	935000 CpGs	Single-base	2017				
Enzyme-based	EM-seq	genome-wide	Single-base	2019	effectively reduces DNA damage, more suitable for trace samples such as cfDNA, more uniform CG coverage, longer library inserts and higher library output	more complex operation flow and longer conversion time	High	Ideal for comprehensive methylation profiling of EC using low-input samples like plasma cfDNA, offering high data quality and accuracy, though its long experimental process and high cost limit suitability for large-scale clinical cohorts
Enrichment-based	MeDIP-seq	genome-wide	Region	2005	the antibody is specific to 5mC	low resolution, antibody batch effects	Moderate	Enrichment of methylated DNA fragments in EC for sequencing, enabling genome-wide methylation mapping, but low resolution restricts its use in single-base methylation marker screening

### 3.1 Techniques for single gene methylation detection

#### 3.1.1 Methylation specific PCR

Methylation specific PCR (MSP), first proposed by Herman in 1996, has become a widely technique for identifying the methylation status within particular regions of target DNA sequences. By treating the target DNA fragment with bisulfite and designing two corresponding primers respectively for the methylation and unmethylation of the target site, the target DNA can be detected at the corresponding site according to its methylation status (50, 51). This technique is highly responsive and discriminating for detecting methylation across nearly all CpG sites within CpG islands. Nonetheless, it can only identify a limited number of CpG sites simultaneously, and the methylation status can only be qualitatively analyzed, not accurately quantified.

#### 3.1.2 MethyLight

EADS et al. developed the MethyLight method based on MSP, which overcomes the limitation that MSP cannot be quantified accurately. The probe fluorescence is read out by real-time fluorescence PCR, and the DNA methylation is quantified by measuring Ct value, which is especially suitable for the detection of rare low frequency methylated DNA segments within a context of abundantly present unmethylated DNA (52, 53). Another variant of MethyLight is the Methylation Sensitive High Resolution Melting (MS-HRM) technique, pioneered by Wojdacz, where PCR amplifies both methylated and unmethylated DNA amplicons. The resulting melting curves for the two types of DNA are markedly distinct. Consequently, the methylation levels of unknown DNA amplicons can be deduced by correlating their melting profiles against a set of standard profiles (54).

#### 3.1.3 Digital methylation-specific PCR

Digital PCR was developed after the first generation of ordinary PCR and the second generation of fluorescence quantitative PCR as the third generation PCR technology. Digital PCR is an end-point method that does not require the absolute quantification of nucleic acid of the standard curve (55). It can divide the droplets by about 20000 or more and randomly distribute the samples to different chambers to ensure that some chambers have one or no nucleic acid. After thermal cycling and reading, absolute quantification is carried out according to Poisson distribution (56). The utilization of digital PCR to the methylation detection of DNA can greatly improve the sensitivity and specificity of detection and achieve absolute quantification. LUO et al. used MethyLight dPCR in their research and compared its performance with the widely used traditional MethyLight PCR, showing that MethyLight dPCR is 25 times more sensitive than traditional MethyLight PCR and can detect NTRK3 in mixed samples with methylation levels as low as 0.032% (57).

### 3.2 Techniques for methylation microarray with pre-designed probes

Methylation microarray enables a comprehensive and quantitative assessment of targeted methylation sites through the genome, while boasting high-throughput capabilities that significantly reduce the cost per sample and providing more accurate and precise methylation measurements independently of read depth. Illumina Infinium HumanMethylation450 BeadChip (450K), Infinium MethylationEPIC BeadChip (850K) and Infinium MethylationEPIC v2.0 BeadChip (935K) are three widely accessible commercially available methylation chips, covering approximate 450000, 850000 and 935000 CpG sites, respectively (25, 58). These methylation microarrays select “Taq” methylation sites in the CGI of promoter, gene body and other functional regions of genes and detect DNA methylation through complementary hybridization and signal amplification (59). Similar to MethyLight, methylation microarrays contain two types of probes, Infinite I and Infinite II, which generate fluorescent signals. Infinium, I consists of U or M of microbeads, matched with unmethylated and methylated sites respectively for single nucleotide extension and generate signals, while Infinite II has only one microbead with tail C, and only a single nucleotide is added during pairing extension. According to the fluorescence color, the type of added base is A or G to determine whether the site is methylated or not (49).

### 3.3 Techniques for genome-wide DNA methylation sequencing

#### 3.3.1 Bisulfite-based conversion technologies

The conversion of genomic DNA through sodium bisulfite treatment to distinguish between unmethylated and methylated cytosines has emerged as the predominant method for DNA methylation analysis, widely adopted across various research and clinical applications. Unmethylated cytosines (C) undergo conversion to uracils (U) after treatment with sodium bisulfite, while 5-methylcytosine (5mC) and 5-hydroxymethylcytosine (5hmC) are shielded from this transformation, thus remaining intact (60). Common bisulfite conversion-based sequencing approaches include whole-genome bisulfite sequencing (WGBS), RRBS, oxBS-seq and methylation microarray. WGBS is considered to be the gold standard of DNA methylation detection, delivering a high-resolution, single-nucleotide map of 5mC across the entire genome (61). It is favored by researchers due to its high conversion rate (>99%), reproducibility, and ease of use through commercial kits. Nevertheless, bisulfite treatment under harsh reaction conditions causes to DNA, resulting in fragmentation, DNA loss and significant GC bias sequencing data (62). In addition, cannot distinguish 5mC from 5hmC. Post-bisulfite adaptor tagging (PBAT) is a variant of WGBS which has been optimized by adapter tagging and two rounds of random primer extension following bisulfite treatment. PBAT is capable of accurately measuring DNA methylation at up to 48.4% of CpGs even at the single-cell level (63). RRBS involves the use of restriction endonucleases to enzymatically cleave the genome, enriching important epigenetic regulatory regions such as promoters and CpG islands and performing bisulfite sequencing. This technique significantly improves the sequencing depth in regions densely populated with CpG sites, enabling the acquisition of highly precise and detailed methylation profiles within CpG islands, promoter regions and enhancer element regions, which is an accurate, efficient and economical method for DNA methylation research and exhibits broad applicability in the study of large-scale clinical samples (64–66). However, RRBS requires high-quality DNA, it is not suitable for the detection of trace and severely fragmented cfDNA. oxBS-seq, in which 5hmC is oxidized to 5-formylcytosine (5fC) with a chemical oxidizing agent while 5mC remains unchanged, can exclude the signal interference of 5hmC on 5mC and achieve the purpose of accurate detection of 5mC (67, 68). Both RRBS and oxBS-seq are based on bisulfite conversion and have therefore inherited the disadvantages of WGBS.

#### 3.3.2 Enrichment based technologies

The principle of affinity enrichment-based methods is using an antibodies raised against 5mC or methyl-CpG binding proteins to selectively capture methylated genomic DNA fragments, which is represented by MeDIP-seq (69). 5hmC (hMeDIP) and 5fC and 5-carboxy cytosine (5CaC) can also be detected by MeDIP-seq with the appropriate antibodies. A modified version of MeDIP for cfDNA detection has also been developed, in which the DNA input amount can decrease to 1–10 ng (70). Compared with WGBS, MeDIP-seq is a suitable and cost-effective approach because of the smaller data and lower cost. However, MeDIP-seq only provides a 150 to 200 bp resolution of the methylome and can’t reach single base resolution (71). Another limitation is that the specificity and selectivity of the methylated antibodies needed are high, and the antibodies are usually more biased to bind to the hypermethylated regions. In addition to MeDIP-seq, other prominent enrichment-based techniques are MBD-seq, relying on methyl-binding proteins MBD2 and MBD3L1 and methylation-sensitive restriction enzyme digestion sequencing (MRE-seq) (72, 73). Integrating MeDIP-seq and MRE-seq can overcome the limitation of MeDIP-seq and enhance the efficiency of the whole genome DNA methylation profiling (74).

#### 3.3.3 Enzyme-based conversion technology

Enzyme-based conversion is a new method for identification of 5mC and 5hmC which is represented by Enzymatic methyl-seq (EM-seq). EM-seq is a two-step enzymatic conversion process for the detection of modified cytosines, utilizing a set of three enzymes including translocation methylcytosine dioxygenase 2 (TET2), T4-phagebeta-glucosyltransferase (T4-BGT) and apolipoprotein B mRNA editing enzyme catalytic subunit 3A (APOBEC3A). The first step involves TET2 to catalyze the cascade oxidization of 5mC to 5hmC, then 5fC, and finally 5caC. Simultaneously, T4-BGT appends a glucose group from UDP-glucose to 5hmC sites in the DNA double helix, resulting in the formation of 5gmC. The synergistic action of TET2 and T4-BGT shields 5mC and 5hmC from the deamination process catalyzed by APOBEC3A. During the subsequent reaction, APOBEC3A converts deoxycytosine to deoxyuracils, yet it does not affect the safeguarded variants of 5mC or 5hmC. The 5mC and 5hmC identified in EM-seq libraries correspond closely to those found in bisulfite-converted libraries (75).

cfDNA methylation is regarded as the best biomarker for predicting the source of cancer signals (33). Compared with other genomic DNA, the content of cfDNA is significantly reduced with high fragmentation and easy to degrade, which puts forward higher requirements for methylation detection technology. EM-seq has several advantages over heavy bisulfite treatment. The EM-seq process is gentler to DNA and reduces DNA damage to a greater extent, and the DNA conversed by EM-seq is more complete, providing libraries with longer inserts and even achieving higher library yields with fewer PCR cycles than WGBS and shows more uniform CG coverage, which can more truly reflect the original sample. Furthermore, EM-seq can detect more CpG sites at lower sequencing depth and has a high correlation between different inputs (75–77). EM-seq is nowadays the most advanced technology to study the whole genome methylation of cfDNA, which has been applied to the diagnostic research on hepatocellular carcinoma and high-risk neuroblastoma with a superior diagnostic performance compared to WGBS (78, 79).

## 4 Computational analysis of methylation data of EC

Single gene methylation detection such as sanger sequencing is one of the earliest methods used to detect DNA methylation status. This approach typically includes enrichment of methylated DNA, sequencing library construction, and subsequent sequencing and data analysis. With this method, researchers can accurately detect the methylation levels of specific genes or genomes, thereby revealing the patterns and regularities of DNA methylation. However, single gene sequencing technologies typically have lower throughput and higher costs, which limits their application in large-scale studies. To overcome these limitations, high-throughput methylation sequencing technology has emerged. High-throughput sequencing technology is an advanced molecular biological analysis method, which represents the frontier of genomics research, can simultaneously sequence a large number of DNA molecules in parallel, with the advantages of high throughput, high sensitivity, low cost and flexibility. In the field of methylation sequencing, high-throughput sequencing technology is widely used in whole-genome methylation analysis, allowing researchers to simultaneously detect the methylation status of the entire genome and obtain more comprehensive and in-depth information.

### 4.1 Analysis under sanger sequencing

Student’s test is the most basic method for comparing methylation levels between the two groups. If the data conform to normal distribution, Student’s test can be used (80). If the data does not satisfy the normal distribution, the Kruskal-Wallis test in the non-parametric test can be used (81). These tests can assess whether the differences in methylation levels between the two groups are significant. When comparing the frequency or proportion of methylation states between different groups, Chi-square tests can be used (82, 83). If the sample size is small and does not meet the conditions of the Chi-square test, Fisher exact test can be used (84). Logistic regression models can also be selected according to the situation, by constructing regression models, it is possible to evaluate the influence of different factors on methylation levels, and calculate the effect size and significance for analyzing the relationship between methylation levels and clinical parameters or other biological variables (85).

### 4.2 Analysis under high throughput sequencing

The examination of differentially methylated regions (DMRs) stands as a pivotal aspect of applied methylation studies, encompassing a comparative evaluation across various genomic segments from an array of samples. The overarching objective lies in the identification of aberrant methylation zones that distinguish neoplastic tissues from their healthy counterparts, with the potential to act as diagnostic indicators or to shed light on the underlying pathological processes. The methodologies for conducting such DMR analyses are diverse, grounded in statistical models and tailored to the specific demands and datasets at hand, with a selection of prominent approaches delineated as follows.

The usual steps for the identification of specific DNA methylation markers for EC are:(i) Obtain DMRs between cancer and healthy DNA. This step is done by comparing the DNA methylation patterns of cancer patients and healthy individuals to identify areas of significant difference between the two. (ii) Obtain DMRs of cancer DNA genomic DNA. By comparing the methylation patterns of DNA genomic DNA in cancer patients, it is possible to further identify cancer-specific DMRs. (iii) Tumor specific DMRs were determined. By comparing the DMRs obtained in the previous two steps, we can find out their common regions, which are tumor-specific DMRs. These regions exhibit specific methylation patterns in the DNA of cancer patients that do not appear in healthy individuals or normal tissues. (iv) A tumor-free methylation profile was used as a negative control. This step is to ensure that the tumor-specific DMRs identified are not caused by non-specific factors but are truly cancer-related.

In high-throughput data, the above generation sequencing data analysis method can also be used to find differential methylation regions or sites. In addition, in the realm of complex and multifaceted methylation data, the application of principal component analysis serves as an effective strategy for dimensionality reduction. This method enhances the interpretability of the data by simplifying its structure, thereby enabling the detection of key methylation signatures that might otherwise be obscured (86). Cluster analysis can group samples with similar methylation patterns to find differences between groups (87). If the actual methylation state of CpG is not taken into account in the predefined region, DMR call methods with variable CpG fragment length are proposed, such as CpG_MPs (88), MOABS (89) and DSS-single (90). With the development of computer technology, some advanced methods have been proposed, such as random forest in machine learning methods, support vector machines (SVM), etc. (91), which can process high-dimensional data and discover complex patterns and interactions. There are specialized analysis packages for differential methylation analysis, such as BSmooth, methylKit, etc. (92) which are specifically designed to process methylation data, including steps such as data preprocessing, quality control, identification of differential methylation regions, and statistical testing.

## 5 Clinical applications and challenges of DNA methylation in early screening and diagnosis of EC

Compared with genetic mutation testing, DNA methylation testing has shown its unique role in cancer diagnosis. First, it is able to capture clinical signals with greater sensitivity and has a wider detection range, thanks to the numerous methylation sites within the tumor and the diversity of CpG sites in each target genomic region (93). Secondly, compared with gene mutations, which are usually highly individual and heterogeneous, it is difficult to accurately indicate the specific source or organ of the tumor (94, 95), while the methylation pattern of DNA can reflect its tissue origin, providing the possibility of tissue tracing (96). Finally, considering that the total number of methylated sites far exceeds the number of gene point mutations, a comprehensive analysis of a set of CpG sites, known as methylated blocks, as a unit can enhance the strength of the signal, thanks to the lateral correlation of these sites and the cumulative effect on the vertical (97).

### 5.1 Early screening diagnostic markers under sanger sequencing technology

Numerous evidences have shown that hypermethylation of the promoter region of tumor suppressor genes is an important event in the development of EC. Therefore, in the context of sanger sequencing, specific tumor suppressor genes were directly selected at an early stage to verify their value as diagnostic markers for EC screening.

*P16* gene is the most common tumor suppressor gene with cell cycle regulation function in human tumors (98, 99). The *p16* gene is considered to be an early tumor diagnostic marker of high value in families at high risk of EC. Mandakini et al. reported that aberrant methylation of the *p16* gene promoter was observed in 81% of the EC patients in their study (100). Additionally, Daito et al. also identified abnormal methylation of the gene promoter region in EC patients (101). It was also found that the hypermethylation of *p16* gene occurred in the continuous process of esophageal epithelial metaplasia and atypical hyperplasia cancer, and its level increased with the increase of the lesion grade (40, 102), suggesting that the degree of methylation of *p16* gene can indicate the degree of progression of precancerous lesions of esophagus, which may have the value of auxiliary diagnosis in population screening.

Somatostatin (*SST*), as a growth hormone release inhibitor and tumor suppressor gene (103), can induce cell apoptosis by blocking the secretion of growth stimulating hormone and growth factor, and directly or indirectly inhibit the occurrence and development of tumors (104). Stephen and colleagues conducted a study investigating the hypermethylation status of the *SST* promoter in 260 esophageal tissues. Their findings suggest that this biomarker has potential utility in differentiating between ESCC, EAC, and normal esophagus, with a high area under the receiver operating characteristic curve of 0.919 (*P < 0.01*) (105). Later, Dai et al. used the Cancer Genome Atlas (TCGA) database to find the differential region of *SST* gene in EC and normal population, and further analysis of tissue samples found that the methylation state of *SST* in tumor tissues was significantly higher than that in neighboring non-cancer tissues, with a sensitivity of 59.3% and a specificity of 72.8% for diagnosis of EC (106). These results provide a basis for *SST* as a potential biomarker for early diagnosis of EC.

*MGMT* is a specific DNA direct reverse repair protein and tumor suppressor gene (107), downregulation of *MGMT* expression through promoter methylation has been shown to occur in many tumors (108, 109). Das et al. compared the methylation ratio of *MGMT* in the blood of 100 patients with ESCC with that of normal controls and found that 70% of the patients had *MGMT* methylation (110). A meta-analysis involving nine clinical cohort studies of EC (861 patients with EAC) showed that *MGMT* promoter methylation was significantly more frequent in cancerous tissues than in paracancer and normal tissues, with OR values of 6.73 and 13.86, respectively (111). Another meta-analysis that included 17 studies (1368 patients with EC and 1489 non-malignant controls) showed that *MGMT* promoter methylation was significantly higher in EC tissue samples than in non-malignant tissue samples (OR 3.64, *P< 0.001*). The results may be related to age, lymph node status, and clinical stage (112). These studies suggest that *MGMT* methylation is a biomarker for EC, which can be diagnosed by testing different types of specimens and gene products.

*HIN-1*, a gene that actively suppresses tumor growth and is abundantly present in a variety of normal epithelial cells (113, 114). According to Guo et al. ‘s study, which revealed that the gene’s methylation status was present in a percentage of esophageal dysplasia cases that escalated with the grade of dysplasia: 31% in grade I, 33% in grade II, 44% in grade III, and reached 50% in EC samples. In contrast, no methylation of *HIN-1* was identified in the control group of normal tissue specimens (115). The deleted in colorectal cancer (DCC) gene is absent in many human cancers, and Park et al. found that DCC methylation was detected in 70% of primary ESCCs but not detected in the normal esophagus of healthy individuals (116). Reprimo, as a candidate tumor suppressor gene, is involved in the regulation of *p53*-mediated G2 phase cell cycle arrest (117), the results showed that the methylation level and frequency of Reprimo in patients with EC were significantly higher than those in normal esophagus, and the AUC was 0.812 (P < 0.00001; 95% confidence interval is 0.73-0.90) (118). The a-kinase anchoring protein 12 (*AKAP12*) has tumor inhibitory activity (119, 120), studies showed that its hypermethylation can significantly distinguish EAC from ESCC and normal esophagus, AUC of 0.943, showing high differential diagnosis ability (P < 0.0001) (121).

In addition, a large number of meta-analysis studies with high-quality evidence have pointed out that additional tumor suppressor genes can be used as promising diagnostic markers for early EC. For example, The runt-related transcription factor 3 (*RUNX3*) gene is a tumor suppressor gene involved in the TGF-β signaling pathway (122), Wang et al. conducted a meta-analysis of 558 patients from 9 eligible studies and found that *RUNX3* methylation was significantly higher in EC than normal squamous mucosa and benign esophageal lesions at the proximal resection margin, and played an important role in the development of EC (123). The *CDH1* gene has been identified as a suppressor of tumorigenesis and is responsible for the production of a transmembrane glycoprotein called e-cadherin, which is predominantly found on the epithelial cell surface (124). Fan et al. included 1633 samples from 13 studies and suggested that high *CDH1* methylation was significantly associated with increased EC risk, and *CDH1* methylation was significantly associated with EC risk (125).

A large number of tumor suppressor genes can be silenced due to hypermethylation of their promoter regions, and the functions of these genes cover important molecular pathways such as cell cycle regulation, apoptosis regulation, cell adhesion and metastasis regulation, and DNA repair (38). All these phenomena indicate that hypermethylation of tumor suppressor genes is an early event in the occurrence and development of EC. Therefore, it is expected that detection of hypermethylation of related tumor suppressor genes can achieve early detection and diagnosis of EC.

In addition to site-specific CpG island promoter hypermethylation, DNA methylation in human cancers is currently known to include global DNA hypomethylation (126). Long interspersed nuclear element-1 (*LINE-1*, L1) makes up a large part of the human genome, so *LINE-1* methylation levels are often used as a measure of global DNA methylation status (127). Studies have shown that the level of *LINE-1* methylation in cancer tissues is significantly lower than that in non-tumor tissues (128). Related mechanism studies have found that *LINE-1* hypomethylation in upper digestive tract cancers may obtain aggressive tumor characteristics through L1 insertion dependent inactivation of tumor suppressor genes (129).

The tachykinin-1 (*TAC1*) gen, a site of frequent heterozygosity loss in EC (130), has been mapped to chromosome 7q21-22 (131). Previous studies by Stephen et al. have found that *TAC1* promoter methylation (132), another study found that *TAC1* hypermethylation in tissue samples could highly distinguish ESCC and EAC from normal esophagus, and the AUC was 0.859(*P < 0.0001*) (133). XIAP-associated factor 1 (*XAF1*) is recognized as a potential tumor suppressor (134), and *XAF1* has been scrutinized for its role in cancer progression, particularly in gastric and colon malignancies (135). In a study by Chen’s team, the methylation profile of *XAF1* was meticulously quantified in both primary EC tissues and their adjacent, cancer-free counterparts. The study revealed a prevalent methylation signature in EC. Furthermore, it was discovered that the expression levels of *XAF1* are influenced by the hypermethylation of its promoter region, indicating a regulatory mechanism in the development of cancer (136). *TFF1* is one of the three Trefoil factors (*TFF*), a protease resistant peptide, which is mainly involved in maintaining the integrity of the gastrointestinal mucosa (137). Isabela et al. ‘s study suggested that *TFF1* methylation levels were elevated in the peripheral tissues of ESCC patients (65 cases) compared with healthy esophagus of non-cancerous individuals (88 cases), and the sensitivity and specificity of *TFF1* methylation to distinguish ESCC patients from healthy esophagus was 78.3% and 90.9% (138).

### 5.2 Early screening diagnostic markers under high-throughput sequencing technology

With the advancement of sequencing technology and analysis methods, in addition to methylation studies of single locus genes, an increasing number of studies have used array data or WGBS data to carry out DMRs analysis. These studies aim to identify specific polygenic methylation sites or regions and develop early screening and diagnosis models for EC. Some studies have also constructed models by summarizing the methylation gene loci found in previous research (**Table 2**).

**Table 2:** Study on early screening and early diagnosis model of esophageal cancer constructed by polygene methylation sites under the background of second-generation sequencing.

Author	Research object	Sample type	Sequencing method	Statistical method	Genes	Key finding
Muhammad et.al (2013) (144)	Discovery: BE (22), EAC (26) Validation: BE (60), EAC (90)	Tissue	27K methylation arrays	Wilcoxon, Pearson’s, ANOVA, Logistic regression	SLC22A18, PIGR, GJA12, RIN2	AUC = 0.988, Sensitivity = 94%, Specificity = 97%
Peng et.al (2017) (156)	EC (43), PAR (43), health (10)	Tissue	450K BeadChip	Heat map	BNIP3, BRCA1, CCND1, CDKN2A, HTATIP2, ITGAV, NFKB1, PIK3R1, PRDM16,PTX3	AUC = 0.988
Pu et.al (2017) (146)	ESCC (94), PAR (94)	Tissue	Targeted bisulfite sequencing	Logistic regression, Machine learning	STK3, cg19396867, cg20655070, ZNF418, ZNF542	AUC = 0.85, Sensitivity = 75%, Specificity = 88%
Tang et.al (2019) (147)	ESCC (74), PAR (74)	Tissue	Pyrosequencing	Wilcoxon, Mann-Whitney U	PAX1, SOX1, ZNF582	AUC = 0.914, Sensitivity = 94%, Specificity = 77%
Qin et.al (2019) (161)	Discovery: EC (28), health (7) Tissue Validation : EC (76),health (17) Plasma Validation : EC (85),health (98)	Tissue, Plasma	whole methylome sequencing, representation bisulfite sequencing	Variance-inflated logistic regression, Recursive partitioning	FER1L4, ZNF671, ST8SIA1, TBX15, ARHGEF4	AUC = 0.93
Li et.al (2019) (145)	Discovery : BE (70), EAC (151), ESCC (60) Validation : BE (32), EAC (76), ESCC (31)	Tissue	450K BeadChip	T-statistics, Least absolute shrinkage, Selection operator	12 CpG (CAPN10, TRIM31, TRIM1S, CLIC1, TACC2, SHANK2, ATP11A, XRCC3)	AUC = 0.992
Sofia et.al (2020) (157)	EC (124), health (56)	FFPE	Targeted bisulfite sequencing	Wilcoxon, Pearson’s, Logistic regression	COL14A1, GPX3, ZNF569	AUC = 0.85, Sensitivity = 69%, Specificity = 96%
Fazlur et.al (2021) (148)	ESCC (108), PAR (51)	Tissue	Targeted bisulfite sequencing	Robust regression, SVA	PAX9, SIM2, THSD4	AUC = 0.98
Qiao et.al (2021) (162)	Discovery: EC (24), PAR (24) Tissue Validation : EC (85), health (125) Plasma Validation : EC (83), health (98)	Tissue, Plasma	Targeted bisulfite sequencing	Fivefold cross-validation, Supporting vector machine	921 DMRs	AUC = 0.932, Sensitivity = 76.2%, Specificity = 94.1%
Ke et.al (2022) (159)	ESCC (93), PAR (120)	Tissue	Targeted bisulfite sequencing	Wilcoxon, Fisher exact test, LASSO regression	cg20655070, SLC35F1, TAC1, ZNF132, ZNF542	AUC = 0.94
Xi et.al (2022) (163)	ESCC (91), PAR (91)	Tissue	450K BeadChip	Wilcoxon, Spearman, Random forest analysis, LASSO regression	HDAC11, MMP13, CPS1, AFF3, LDB2, HOXC10, PDE4D, SYNE3, ZNF578, YEATS2, 5'UTR, PACRG, SLC8A3	AUC = 0.971, Sensitivity = 96.7%, Specificity = 100%
Pei et.al (2023) (155)	Discovery : EC (48), health (101) Validation : EC (20), health (20)	Plasma	Quantitative methylation-specific PCR	Multinomial logistic regression	ZNF582, FAM19A4	AUC = 0.675, Sensitivity = 60.4%, Specificity = 83.2%
Dai et.al (2023) (154)	Tissue Discovery: EC (10), PAR (10) Plasma Discovery: EC (11), health (57) Plasma Training : EC (96),health (51) Plasma Validation : EC (82),health (75)	Tissue, Plasma	Targeted bisulfite sequencing	Mann-Whitney U, Pearson’s, Logistic regression	KCNQ5, C9orf50, CLIP4, ELMO1,ZNF582, TFPI2	AUC = 0.937

*PAR, Indicates adjacent cancerous tissue.

Firstly, Muhammad et al. developed a four-gene model that can effectively distinguish between Barrett’s esophagus and EAC patients, achieving an AUC of 0.988 with a sensitivity of 94% and specificity of 97% (139). Li et al.’s diagnostic model based on 12 CpG sites also achieved a high AUC value of 0.992 in distinguishing between these two conditions (140). Secondly, numerous studies focused on constructing diagnostic models using ESCC samples and adjacent tissues. These models demonstrated superior diagnostic performance with generally higher AUC values than 0.95 (141–144). Thirdly, some diagnostic models for EC transitioned from tissue samples to plasma samples as the sample type. The initial marker screening was performed using tissue samples, but subsequent construction of the diagnostic model utilized plasma data from both EC patients and healthy controls. These plasma-based models exhibited high performance with AUC values generally exceeding 0.90 (145–147). Finally, there were studies that solely used plasma samples from patients for marker screening and determination resulting in lower performance levels for the constructed EC diagnosis model with an AUC reaching only up to 0.675 (148). Therefore, although the multigene methylation diagnostic model of EC based on plasma samples has the advantages of minimally invasive, efficient and cost-effective, the determination of its markers and its performance need to be further optimized in the future.

### 5.3 Validated DNA methylation biomarkers in EC

Recent studies have validated DNA methylation biomarkers for early detection and risk stratification in EC and its precursor lesions. One notable study (149) introduced a nonendoscopic technique using a sponge-capsule device to identify Barrett’s esophagus (BE), high-grade dysplasia (HGD), and esophageal adenocarcinoma (EAC) through methylated DNA markers. The study pinpointed 12 methylation markers that were significantly over-methylated in BE compared to normal esophagus tissue. A 3-gene panel (*USP44*, *TBC1D30*, and *NELL1*) effectively differentiated HGD or EAC from normal controls, achieving an AUC of 0.911 in the training cohort and 0.969 in the validation cohort. Another significant study (150) unveiled Esopredict, a methylation-based assay designed to assess the risk of neoplastic progression in BE patients. The assay revealed that high-risk patients had a 15.2-fold higher likelihood of progressing to HGD or EAC within five years compared to low-risk patients. The average risk of progression to HGD or EAC within five years was 21.5% for high-risk patients, markedly higher than the 1.85% risk observed in low-risk patients. Further validation studies (151) demonstrated that Esopredict maintains high sensitivity across various spatial and temporal sampling points. In a cohort of 52 spatially profiled patients, Esopredict achieved a sensitivity of 81% based on the highest-scoring biopsy from each patient, which increased to 100% when endpoint biopsies were taken within five years of the initial biopsy. In a group of 28 temporally profiled patients, the sensitivity was 100% based on the biopsy closest to the endpoint biopsy. These findings underscore the potential of DNA methylation biomarkers for noninvasive screening and personalized management of esophageal cancer, offering promising new avenues for early detection and risk stratification.

### 5.4 Ongoing or completed DNA methylation clinical trials

According to data from clinical trial centers in China and the United States, several experiments have been conducted for DNA methylation-based early screening and auxiliary diagnosis of EC. These include research on MT-1A, Epo, and Septin9 gene methylation for auxiliary diagnosis at the Cancer Hospital of the Chinese Academy of Medical Sciences (ChiCTR2400083525), which involved a model-verification cohort of 297 participants and a clinical validation cohort of 1429 participates. The combined model of these three methylation markers achieved 85.5% sensitivity and 95.3% specificity for EC detection, with notably higher sensitivity for early-stage tumors (56% for stage 0 and 77% for stage I) compared to conventional biochemical markers. Moreover, in high-risk populations for EC, the panel achieved a sensitivity of 85.86% and a specificity of 93.61% (152). Notably, a study by the First Affiliated Hospital of Naval Medical University evaluated a cfDNA methylation assay (IEsohunter) targeting OTOP2 and KCNA3 for non-invasive detection of EC. Among 1116 participants, the IEsohunter test achieved 87.4% sensitivity and 93.3% specificity for EC, with sensitivity increasing from 78.5% in stage I to 96.9% in stage IV and turned negative after surgical resection of EC (153). Additionally, ongoing clinical trials are exploring DNA hydroxymethylation for early screening of EC at Nanfang Hospital of Southern Medical University (NCT03922230). These studies collectively highlight the potential of DNA methylation markers as effective tools for early detection and surveillance of EC. Furthermore, there have been promising results in DNA methylation-based early screening diagnostic tests for multiple cancers including EC. For instance, Grail’s PATHFINDER Real-world Cohort Study (NCT04241796) developed a multi-cancer screening model with 99.1% specificity, 98.6% NPV, and 97% traceability accuracy (154). The THUNDER cohort study by Burning Rock Biotech (NCT04820868) demonstrated a sensitivity of 69.1%, specificity of 98.9%, and tissue tracing accuracy of 91.7% in the screening and diagnosis of six types of cancer including EC and lung cancer (155). Singlera’s TZL longitudinal cohort study showed that their PanSeer model achieved a sensitivity of 95% and specificity of 96% in the early screening for five types of cancer including colorectal cancer and EC (156). Moreover, several large cohort studies focusing on EC are currently underway such as Grail’s NHS-Galleri and Gallery-Medicar cohorts which involve over 140,000 individuals in the UK as well as 50,000 individuals in the US aiming to conduct early screenings for various cancers among healthy populations. Burning Rock Biotech’s PREDICT and PRESCIENT also contribute to this field through more than10,000 prospective blind validation cohorts utilizing DNA methylation, serum tumor markers and RNA combined models to facilitate advancements in early screenings, to reveal the difference of molecular characteristics of cancer patients with different etiology and pathology.

### 5.5 Commercial applications of DNA methylation biomarkers in EC

The clinical relevance of DNA methylation biomarkers has led to the development of several commercial products aimed at improving the early detection and diagnosis of EC. One such product is “Doctor Si^®^”developed by BioChain which is the first esophageal cancer methylation detection kit approved by the National Medical Products Administration (NMPA) in China. This detection kit utilizes three DNA methylation markers to identify early-stage EC and has a sensitivity of 85.5% and a specificity of 95.3%, making it a valuable tool for early screening, especially for patients who refuse endoscopy (152). Another detection kit developed by Ammunition Life-Tech Company has an overall sensitivity of 87.4% and specificity of 93.3% for EC detection, with a sensitivity of 51.35% for high-grade intraepithelial neoplasia (HGIN) (153). GRAIL’s Galleri test achieves 93.7% accuracy in tissue-of-origin prediction for EC signals (33), while Burning Rock’s ELSA-seq technology shows 74.7% sensitivity at 95.9% specificity in multi-cancer detection including EC (146). These commercial products offer a standardized and accessible method for clinical application, potentially enhancing the early detection rates and improving patient outcomes. However, the widespread adoption of such products requires rigorous validation and cost-effectiveness studies to ensure their practicality in routine clinical settings.

## 6 Future directions

The application of DNA methylation biomarkers in EC screening and diagnosis holds significant promise for improving early detection and patient outcomes. High-throughput methylation profiling advances have facilitated the identification of numerous methylation markers associated with EC, offering potential for non-invasive testing modalities (155). Liquid biopsy, which examines DNA methylation patterns in blood samples, is emerging as a potent tool for early cancer detection, including EC (157).

Emerging evidence suggests significant ethnic variability in EC methylation patterns, which could influence the effectiveness of DNA methylation-based biomarkers. For instance, *RASSF1A* hypermethylation in the Chinese cohort was much lower than that in Japanese ESCC patients (52% vs 74%). Future research should consider these variations to ensure the broad applicability of such biomarkers across different populations (158).

Looking ahead, we anticipate the development of multi-marker panels that integrate methylation signatures with other molecular alterations to enhance specificity and sensitivity (159). The integration of machine learning algorithms could further refine the predictive accuracy of these panels by analyzing complex methylation patterns and their correlation with disease progression (160). Moreover, longitudinal studies are needed to establish temporal dynamics of methylation changes during esophageal carcinogenesis. This could reveal the identification of early methylation alterations that precede tumor formation, creating critical intervention windows before clinical manifestation.

The cost-effectiveness of DNA methylation-based screening tests is a critical consideration for their widespread adoption in clinical practice. Recent advancements in sequencing technologies have significantly reduced the cost of DNA methylation analysis, making these tests more accessible for large-scale screening programs. For instance, NGS technologies have revolutionized the field by offering high-throughput capabilities at a fraction of the cost compared to first-generation sequencing methods. This reduction in cost has been pivotal in making methylation-based screening tests more feasible for routine clinical use. However, the cost-effectiveness is not solely determined by the sequencing technology itself. Other factors, such as sample collection methods, laboratory processing, and data analysis, also play significant roles. For example, the use of liquid biopsy for sample collection is less invasive and more cost-effective compared to traditional tissue biopsies, which require endoscopic procedures. Liquid biopsy allows for the analysis of ctDNA in blood samples, reducing the need for more invasive and expensive procedures.

Moreover, the development of targeted methylation panels has further enhanced the cost-effectiveness of these tests. These panels focus on specific methylation markers that are highly indicative of esophageal cancer, reducing the overall cost of sequencing while maintaining high diagnostic accuracy. For instance, studies have shown that targeted methylation panels can achieve high sensitivity and specificity for detecting esophageal cancer, making them a cost-effective alternative to whole-genome sequencing (WGS) (152, 153). In addition, the integration of machine learning algorithms can further enhance the predictive accuracy of methylation-based screening tests, potentially reducing false positives and improving overall cost-effectiveness. Machine learning models can analyze complex methylation patterns and their correlation with disease progression, leading to more accurate and reliable diagnostic results (160).

In conclusion, DNA methylation has the potential to revolutionize the early detection of EC and the cost-effectiveness of DNA methylation-based screening tests is influenced by multiple factors, including advances in sequencing technology, sample collection methods, targeted methylation panels, and the integration of machine learning algorithms. These advancements collectively contribute to making methylation-based screening tests a viable and cost-effective option for early detection and management of esophageal cancer. Future research should focus on optimizing these factors to further reduce costs and improve the accessibility of these tests in clinical practice.

## 7 Conclusions

In summary, DNA methylation has emerged as a promising biomarker for the screening and diagnosis of EC. The aberrant methylation patterns observed in EC cells provide a unique opportunity for the development of non-invasive diagnostic tests that could facilitate early detection and improve prognosis. The specificity of methylation changes to certain genes associated with esophageal carcinogenesis allows for the potential creation of targeted assays that can detect the presence of cancerous cells with high accuracy.
